# Modeled temperature dependencies of macromolecular allocation and elemental stoichiometry in phytoplankton

**DOI:** 10.1016/j.csbj.2021.09.028

**Published:** 2021-09-28

**Authors:** Gabrielle Armin, Keisuke Inomura

**Affiliations:** Graduate School of Oceanography, University of Rhode Island, Narragansett, RI, United States

**Keywords:** Quantitative model, Nutrients, Macromolecules, Temperature, Photosynthesis, Biosynthesis, Metabolism, Phytoplankton, Climate

## Abstract

Warming oceans may affect how phytoplankton allocate nutrients to essential cellular processes. Despite the potential impact of such processes on future biogeochemical cycles, questions remain about how temperature affects macromolecular allocation and elemental stoichiometry within phytoplankton cells. Here, we present a macromolecular model of phytoplankton and the effect of increasing temperature on the intracellular allocation of nutrients at a constant growth rate. When temperature increases under nitrogen (N) and phosphorus (P) co-limitation, the model shows less investment in phosphorus-rich RNA molecules relative to nitrogen-rich proteins, leading to a more severe decrease in cellular P:C than N:C causing increased cellular N:P values. Under P limitation, the model shows a similar pattern, but when excess P is available under N limitation, we predict lowered N:P due to the effect of luxury uptake of P. We reflected our model result on the surface ocean showing similar latitudinal patterns in N:P and P:C to observation and other model predictions, suggesting a considerable impact of temperature on constraining the elemental stoichiometry in the ocean.

## Highlights

• Phytoplankton use and allocate nutrients from their environment to pools of macromolecules to survive.
• We model the effect of temperature to macromolecular allocation in phytoplankton.
• Increasing temperature favors investment to N-rich proteins rather than P-rich RNA molecules.
• Carbon storage increases with increasing temperature, raising the potential for carbon export.
• Our model can be implemented into ocean models to evaluate the impact to global biogeochemical cycles.

## Graphical abstract

**Figure:**
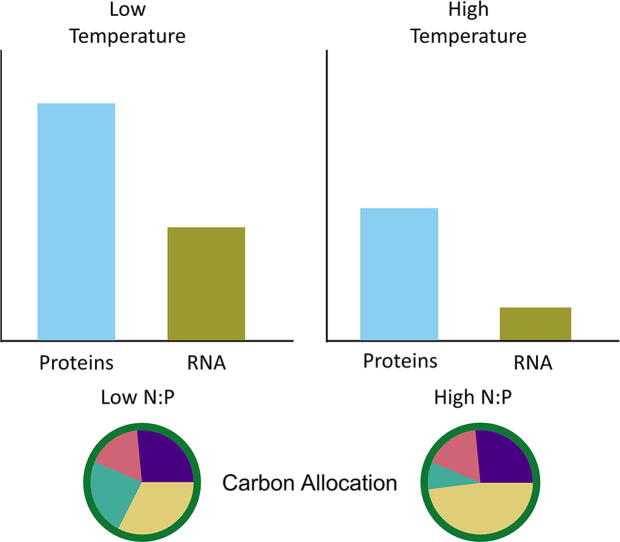

## 1 Introduction

Phytoplankton are key players in global biogeochemical cycles and climate regulation [1], [2]. They consume nutrients under differing environmental conditions, resulting in various elemental ratios (C:N:P) within the cell [3]. The similarity between the elemental composition of phytoplankton cells and the deep-ocean nutrient availability was first described with the Redfield ratio [4], [5]. The ratio was often assumed as stable, but laboratory studies have showed a significant deviation from this elemental ratio [6], [7], [8], [9], which can lead to the large-scale variations in marine organic matter [10], [11], [12]. Determining how environmental factors control this ratio is central to understanding global biogeochemical cycles and the resultant climate due to the considerable impact elemental ratios of phytoplankton have on the export of nutrients to the deep ocean [13], [14], [15].

One of the major influences on cellular elemental stoichiometry is temperature [16]. A recent study shows the optimum N:P supply (defined as N:P ratios that lead to maximum biomass in culture) of phytoplankton (*Chlamydomonas reinhardtii*) increases with temperature, suggesting the effect of temperature on the optimum ratios is similar to the cellular elemental stoichiometry [16]. Also, a model describing allocation of macromolecules, which includes the effect of temperature, predicts a global pattern of N:P in the ocean [17], [18]. However, the effect of temperature on the carbon-related elemental stoichiometry (N:C and P:C) has not been well characterized. Additionally, it is not clear how different nutrient limitations affect the pattern of temperature dependence on elemental stoichiometry.

Recently, Inomura et al. developed a coarse-grained model of phytoplankton (Cell Flux Model of Phytoplankton: CFM-Phyto) [19] and has used the model to estimate the C:P ratio of phytoplankton in the ocean [20]. Conceptually, the model combines physiological acclimation of phytoplankton on elemental stoichiometry [21], [22], coarse-grained macromolecular allocations (e.g., proteins, DNAs, RNAs, carbohydrate, chlorophyll) [23], [24], [25], [26], and broad-brush proteomics informed from recent proteome studies [27], [28], [29]. By quantifying the intracellular macromolecular allocation under various growth conditions (nutrient limitation, growth rate, and light), CFM-Phyto predicts the elemental stoichiometry of phytoplankton.

In this study, we further develop the model resolving the temperature dependence on the metabolisms, macromolecular allocation, and elemental stoichiometry to address the following questions: (1) How does macromolecular allocation vary with temperature at a specific growth rate? (2) How do these variations influence C:P and C:N at a specific growth rate? (3) How does temperature dependence on elemental stoichiometry differ under variant nutrient limitations at a specific growth rate? In this study, we use recent data of the phytoplankton species, *Chlamydomonas reinhardtii*, which provides a clear pattern of the optimum N:P supply and the temperature to constrain the model [16]. The model provides predictions and testable hypotheses addressing the above questions, which we hope will guide future laboratory studies, promoting hypothesis-driven research in computational and experimental biology [30], [31].

## 2 Methods

**Fig. 1:**
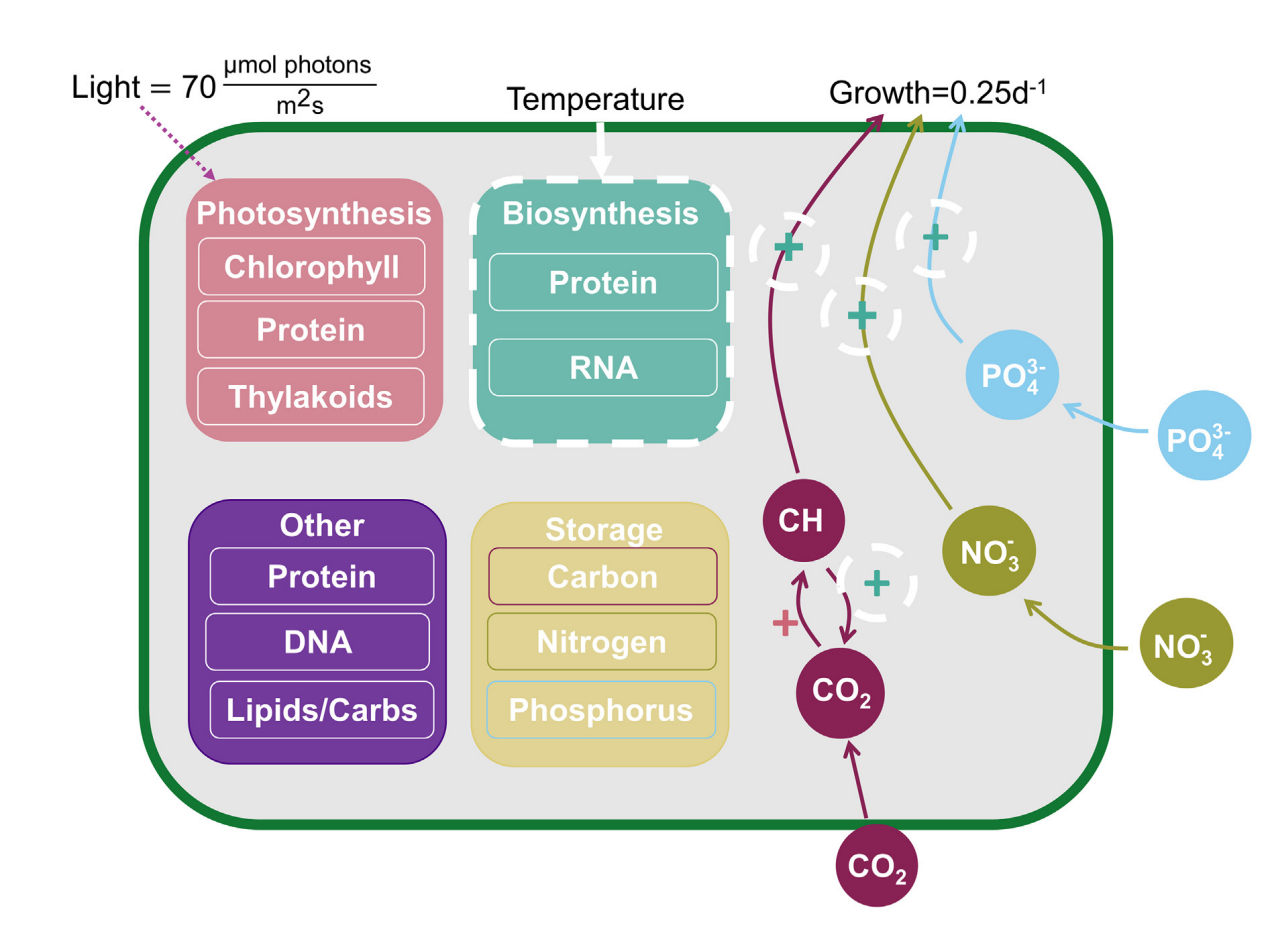
Cell model schematic of phosphorus (blue circles), nitrogen (green circles), and carbon (maroon circles) nutrient fluxes and allocation to basic cellular functions (rounded squares). Our model groups macromolecules into four pools: photosynthetic molecules, biosynthetic molecules, essential molecules for cell survival and structure (labeled as “Other”), and nutrient storage. Photosynthesis positively affects carbon fixation (pink plus), while biosynthesis positively influences cellular respiration and growth (teal pluses). White dashed lines denote processes to which we added temperature effects in the model. The model simulation operated in a co-limiting nutrient scenario with the continuous dilution rate 0.25 day^−1^ and constant light intensity 70 μmol photons m^-2^s^−1^ during the light period of a 12 h:12 h light:dark cycle in accordance with experimental conditions [16]. (For interpretation of the references to colour in this figure legend, the reader is referred to the web version of this article.)

The CFM-Phyto is an idealized model of phytoplankton that predicts steady-state macromolecular allocation under various growth rates, light intensities, and nutrient availability [19]. Several major empirically-supported assumptions form the basis of this model. First, photosynthesis is represented by a saturating function of irradiance [21], [32] and the composition of photosynthetic machinery is fixed [33], [34], [35], [36]. Next, the number of biosynthetic proteins linearly increases with growth rate [27], [28], and there is a linear relationship between RNA and the total protein content and growth rate [24], [37]. In this study, we also assume the general pattern of the elemental stoichiometry is conserved across taxa, which is supported by a compilation of data across species [19]. We divided nutrients amongst various macromolecular pools including photosynthesis, biosynthesis, essential molecules, and storage (Fig. 1). Photosynthetic proteins, chlorophyll, and thylakoid membranes make up the photosynthetic macromolecular pool, whereas the biosynthetic macromolecular pool consists of biosynthetic protein and RNA which is grouped in this pool due to its key function in protein synthesis. Essential molecules include DNA, lipids, and sugars necessary for maintaining cellular structure and basic functions for cell survival. These mathematical relationships coupled with mass conservation and allocation create CFM-Phyto. Healey’s chemostat experiment with the freshwater phytoplankton species, *Synechoccus linearis,* provided elemental stoichiometry constraints for various environmental conditions within the model [38]. Although the model relied on *S. linearis*, the broad trend in macromolecular allocations and elemental stoichiometry are shared across taxa, as seen when tested [19] with two marine species *Pavola lutheri* [39] and *Skeletonema costatum* [40]. The model also captures the broad patterns of growth rate dependencies in protein *Synechocystis* sp. PCC 6803 [27] and other macromolecular allocations in *Prochlorococcus marinus* PCC 9511 [25].

To model the temperature effect within the cell, the light intensity (70 μmol of photons m^−2^ s^−1^ during the light periods of a 12 h:12 h light:dark cycle) and growth rate (dilution of 0.25 d^-1^) were held constant and chosen to align with conditions in a semi-continuous culture [16]. Likewise, we altered key parameters specific to the freshwater species investigated, *Chlamydomonas reinhardtii.* An assumption that more mitochondria [41] are present compared to *S. linearis* led us to assume the initial RNA in the cell is higher, since mitochondria contain their own DNA and RNA molecules [42]. Additionally, we increased the minimum phosphorus level to account for the changes in cellular RNA and ribosome [43] concentration. Lastly, we decreased the stoichiometric ratio of photosynthetic nitrogen to chlorophyll [19], [44] (See supplementary material). We added temperature to the model by applying the Arrhenius equation [45] (Eq. (1)) to quantitative expressions of biosynthesis. We excluded temperature dependence from the photosynthetic machinery, as empirical evidence suggests investments to the photosynthetic machinery are largely independent of temperature [46]. To test other temperature formulations, we ran a simulation using the Q10 formulation (see Fig. S1) and obtained nearly identical results using a Q10 value of 2.8.

(1) $$\mathrm{Arr}=exp\left(\left(-\frac{E_{a}}{R}\right)\times \left(\frac{1}{T}-\frac{1}{T_{\mathrm{ref}}}\right)\right)$$

We set the activation energy ($E_{a}$) to be 70,000 joules (J) per mole (mol) [47], the universal gas constant (R) is 8.3 Jmol^-1^K^−1^, and the reference temperature ($T_{\mathrm{ref}}$) to be 293 Kelvin (K) [38]. We incorporated the resulting rate constant (Arr) into computational representations of biosynthesis rates associated with cellular respiration and growth. Specifically, we divided the constant factor, $A_{\mathrm{Bio}}$, which relates growth rate (μ) to biosynthetic protein, by Arr (Eq. (1)). We used this parameter to estimate the varying dedication to biosynthetic protein $(Q_{C}^{\mathrm{Pro}-Bio}$) with increasing temperature (Eq. (2)).

(2) $$Q_{C}^{\mathrm{Pro}-Bio}=\frac{A_{\mathrm{Bio}}}{\mathrm{Arr}}\times \mu$$

Similarly, we divided the constant factor, $A_{\mathrm{RNA}}^{P}$, which relates growth rate (μ) and protein ($Q_{c}^{\mathrm{Pro}}$) to RNA, by Arr determined in (Eq. (1)). This parameter gave the variations in the investment to RNA ($Q_{P}^{\mathrm{RNA}}$) with increasing temperature (Eq. (3)).

(3) $$Q_{P}^{\mathrm{RNA}}=\frac{A_{\mathrm{RNA}}^{P}}{\mathrm{Arr}}\times \mu \times Q_{c}^{\mathrm{Pro}}+Q_{P,min}^{\mathrm{RNA}}$$

The supplementary material contains a full list of all equations affected by the above changes in the model and descriptions of all parameters used (Tables S2 and S3). The model operates in three nutrient scenarios including co-limiting nutrients (N and P), nitrogen-limited, and phosphorus-limited. The non-limiting nutrient is assumed abundant.

To reflect the model results on the temperature distribution of the surface ocean, we used the temperature data from World Ocean Atlas [48]. The data are based on the statistical mean for years 1955–2017 with the resolution of 5°×5°. We chose this coarse-grained resolution to reduce the computational load. We ran CFM-Phyto for each grid cell based on the local temperature. In this exercise, to isolate the effect of temperature, we assumed a constant cellular growth rate of 0.25 d^-1^ and a saturated light intensity of 1000 μmol of photons m^−2^ s^−1^. To qualitatively compare with a previous model [17], we assumed no N and P storage. We also ran the simulation with increased temperature by 4 °C, similar to the surface temperature increase in 100 years according to the IPCC’s Representative Concentration Pathway (RPC) 8.5 [27], [49].

## 3 Results and discussion

### 3.1 Co-limitation

**Fig. 2:**
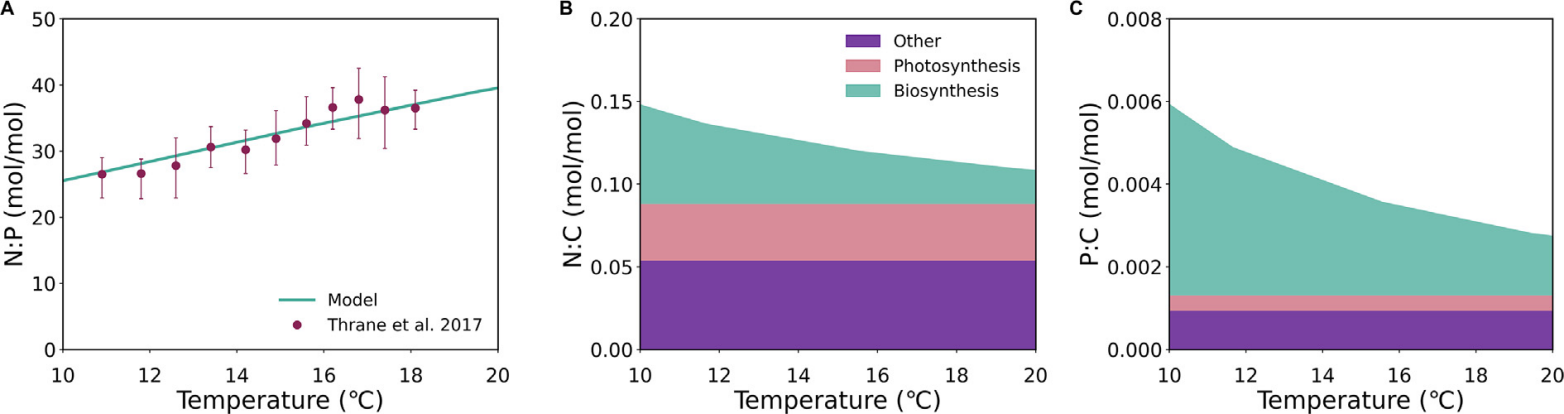
Modeled molar elemental ratios (N:P, N:C, P:C) under nutrient co-limitation over a range of temperatures in Chlamydomonas reinhardtii. Model produced N:P ratios (Panel A) were comparable (R^2^ = 0.95) to data of optimum N:P resource ratio [16]. Error bars indicate upper and lower values of N:P at each temperature [16]. N:C (Panel B) and P:C (Panel C) ratios also include respective N and P allocation to macromolecular pools of biosynthesis (teal area), photosynthesis (pink area), and other (purple area) over the temperature range. (For interpretation of the references to colour in this figure legend, the reader is referred to the web version of this article.)

Our model shows increasing N:P with temperature under N and P co-limitation (Fig. 2A). As temperature rises, the N:P ratio increases with a relative positive change of 54 percent. With the above parameterizations, the modeled cellular N:P ratios for *Chlamydomonas reinhardtii* under co-limitation have a strong correlation (R^2^ = 0.95) to experimental data of optimum resource N:P ratios (defined as N:P ratios that leads to maximum biomass in culture), for increasing temperature [16] (Fig. 2A). We made this model-data comparison for two reasons. First, under the N and P co-limiting situation, the nutrients would be used optimally to maximize biomass without any allocation to N or P storage [16], [19]. Second, it has been suggested that, under steady-state co-limitation, the nutrients are fully consumed, equating the resource and biomass N:P ratios [16], [19], [38].

The temperature variation for N:P is largely explained by the different responses of the nitrogen-rich molecules and the phosphorus-rich molecules. As temperature increases, the number of proteins decreases and the protein production per RNA increases in order to make RNA more efficient. Both nutrient ratios (N:C and P:C) decrease with increasing temperature for this reason (Fig. 2B-C). However, the intracellular level of phosphorus is more strongly affected by temperature than intracellular nitrogen, leading to the observed change in N:P with temperature.

The intracellular level of nitrogen (Fig. 2B) reduces by a quarter over a 10⁰C increase (initial N:C = 0.147; final N:C = 0.108). This moderate decrease with temperature occurs because increasing temperature enhances enzymatic efficiencies [50], [51], [52] and lowers the molecular requirement to achieve a certain growth rate (Fig. 2B). The model simulates this effect for the biosynthetic molecules. Since we hold growth rate constant in this simulation, the increasing temperature lowers the requirement of these molecules due to the increase in metabolic efficiency [53] and the number of biosynthetic molecules is inversely affected by the increased efficiencies with temperature (∼1/*Arr*).

Compared to nitrogen, intracellular phosphorus decreases more severely with temperature (Fig. 2C); P:C reduces to approximately half of the original value over a 10⁰C increase (initial P:C = 0.0058; final P:C = 0.0027). The variation in phosphorus is mainly represented by RNA, which is largely responsible for protein synthesis and proportional to the amount of protein when the growth rate is constant [19], [24]. The demand of RNA decreases more strongly than proteins due to the combination of the following two effects. First, increasing temperature increases the efficiency of protein synthesis per RNA. Second, since the amount of protein decreases with temperature, RNA decreases accordingly. This is consistent with the translation-compensation hypothesis [54], which states as temperature increases, lower ribosomal density is required to maintain the same level of protein synthesis. Here, we term this effect “PRT effect” (protein/RNA temperature effect) for convenience. Since the P:C relative decrease is larger compared to N:C, N:P increases with temperature (Fig. 2).

### 3.2 Phosphorus limitation

**Fig. 3:**
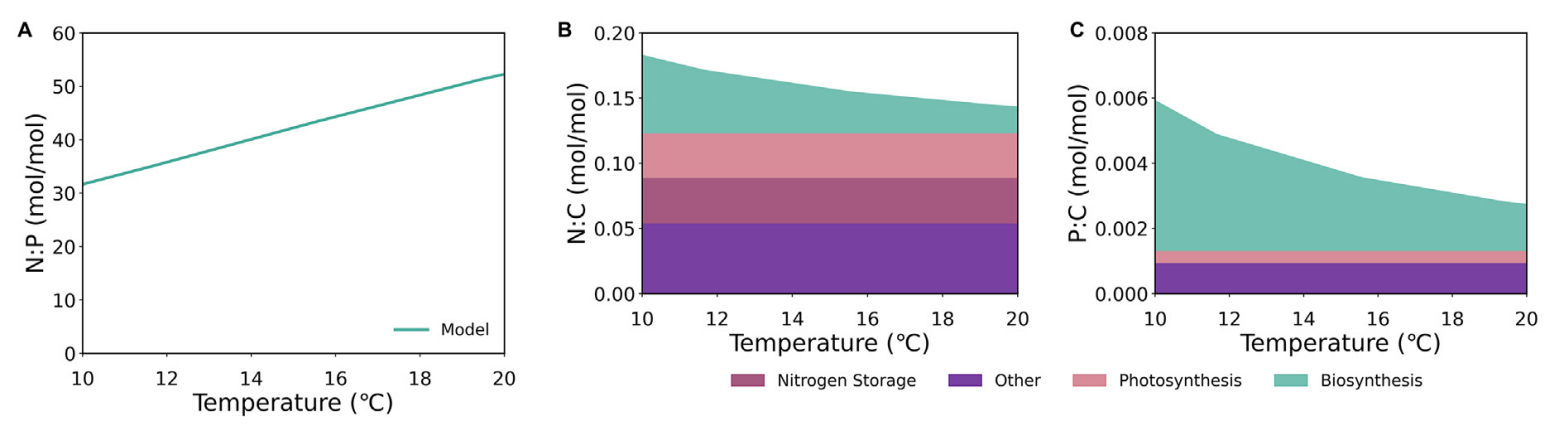
Modeled molar elemental ratios N:P (Panel A), N:C (Panel B), P:C (Panel C) under phosphorus limitation over a range of temperatures. N:C (Panel B) and P:C (Panel C) ratios also include respective N and P allocation to macromolecular pools of biosynthesis (teal area), photosynthesis (pink area), nitrogen storage (maroon area), and other (purple area) over the temperature range. (For interpretation of the references to colour in this figure legend, the reader is referred to the web version of this article.)

To test the effect of excess nitrogen availability, we ran a simulation under phosphorus limitation. Similar to co-limitation, the N:P ratio (Fig. 3A) trend increases with increasing temperature due to the PRT effect. However, the positive relative change (64.3 %) is larger under phosphorus limitation.

The positive shift in the range is due to the addition of nitrogen storage as a macromolecular pool in the model, but the overall macromolecular allocation of nitrogen (Fig. 3) highly resembles the pattern under co-limitation, the only difference being higher values of N:C. The ratio of P:C remains unchanged (Fig. 3C) from the prior condition of co-limitation and follows the same pattern and dedication to three major macromolecular pools. For this reason, N:P ratios increase with increasing temperature for both co-limitation and phosphorus limitation. However, since the nitrogen storage decreases the relative change in N:C, the N:P range shifts positively under phosphorus limitation, reflecting the change in P:C more strongly.

### 3.3 Nitrogen limitation

**Fig. 4:**
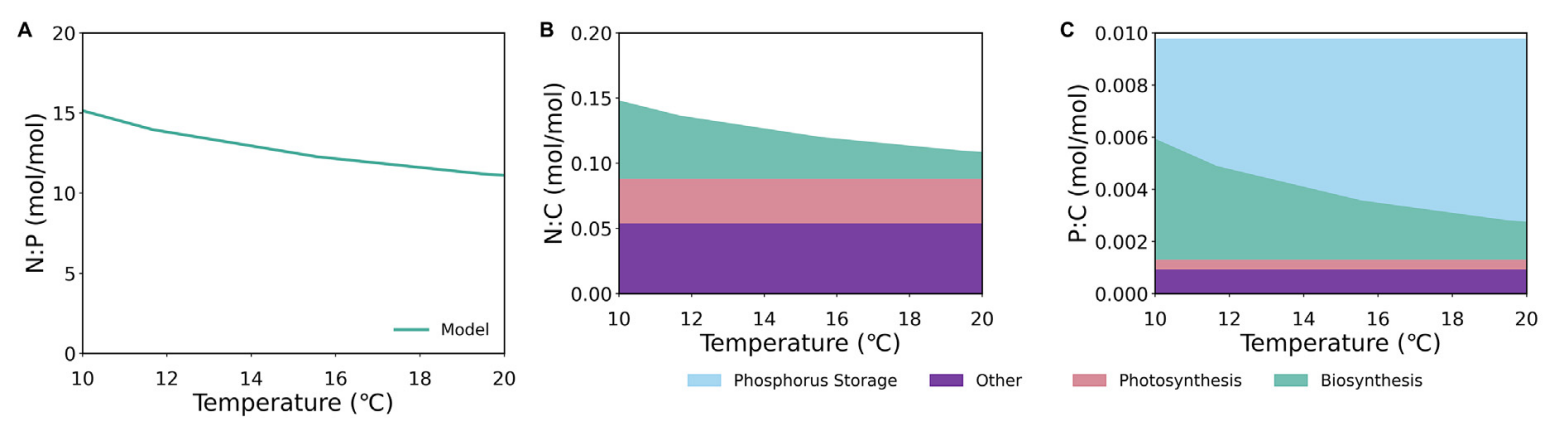
Modeled molar elemental ratios N:P (Panel A), N:C (Panel B), P:C (Panel C) under nitrogen limitation over a range of temperatures. N:C (Panel B) and P:C (Panel C) ratios also include respective N and P allocation to macromolecular pools of biosynthesis (teal area), photosynthesis (pink area), phosphorus storage (blue area), and other (purple area) over the temperature range. (For interpretation of the references to colour in this figure legend, the reader is referred to the web version of this article.)

When nitrogen is the limiting nutrient, the N:P ratio trend changes from previous nutrient scenarios due to phosphorus storage. Under nitrogen limitation, the trend of N:P ratios decreases with a negative relative change of 27 percent (Fig. 4A).

Here, the N:C ratio remains unchanged from the co-limitation scenario (Fig. 4B). The P:C ratio is a constant value, 0.0098, spanning this temperature range due to phosphorus storage (Fig. 4C). This constancy in P:C is supported by the observations of constant P:C under various growth rates for multiple taxa of phytoplankton grown in nitrogen limited environments [19], [38], [55], [56]. The allocation to macromolecular pools of photosynthesis and biosynthesis follows the patterns of previous nutrient scenarios. In this case, the decreasing N:C and constant P:C lead to the decreasing N:P ratios with increasing temperature.

### 3.4 Varying growth rates and light intensities

Our model simulations for different growth rates and light intensities create an understanding of the elemental stoichiometry and nutrient allocation in phytoplankton in vertical oceanic water columns. Higher light intensity produces lower elemental ratios of N:C and P:C (Fig. S2), but have a similar range of N:P, under nutrient co-limitation, compared to low light intensity. Low light intensity elevates the demand for light-harvesting machinery in the photosynthetic macromolecular pool, increasing the requirement for nitrogen-rich molecules and, to a lesser extent, phosphorus-rich molecules.

At the surface of the oligotrophic ocean, nutrients are limited, resulting in slow growth. The model shows that a slower growth results in lower elemental ratios of N:C and P:C (Fig. S3). On the other hand, at greater depths, more nutrients are available and the range of N:P would shift negatively with a higher growth rate under co-limitation. The nutrient requirement of photosynthetic and biosynthetic machinery is higher with a faster growth rate in order to maintain a higher level of cellular growth. This increase in machinery requires more phosphorus-rich molecules, which accounts for the negative shift in N:P ratios.

### 3.5 Increased C storage with increased temperature

**Fig. 5:**
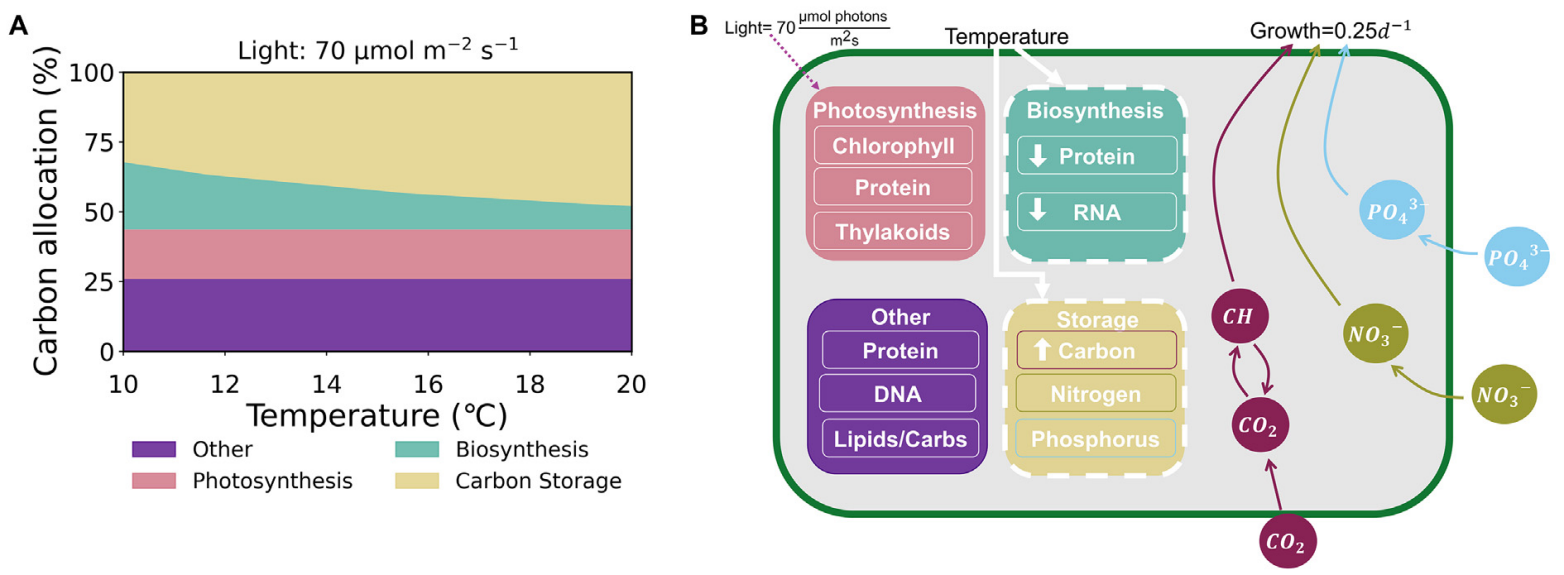
Carbon allocation and summary of the temperature’s effects on the macromolecular allocation. (A) Carbon allocation percentage over a range of temperatures to four macromolecular pools: photosynthetic molecules, biosynthetic molecules, essential molecules (“Other”), and carbon storage. (B) The simulation was under constant growth rate and light intensity for nutrient co-limitation. Cell model schematic with the direct effect of temperature on individual components of each macromolecular pool. White downward arrows represent a decreased allocation with increasing temperature. White upward arrows represent increased allocation with increasing temperature. Constant values do not exhibit any arrows.

Our results indicate when temperature increases, more carbon is stored relative to the limiting nutrient. Specifically, there is an increase in allocation to carbon storage (Fig. 5A) and decreased N:C and P:C ratios (Fig. 2B/C), or rather, increased C:N and C:P ratios, with increasing temperature. These are due to increased efficiencies of molecules, reducing their quantitative needs, allowing more carbon to be allocated to carbon storage or non-functional carbon (Fig. 5). Changes such as these in phytoplankton stoichiometry could ultimately increase the efficiency of the biological pump [20], [57], [58], [59]. Following Broecker’s mathematical representation [13], carbon export is proportional to the uptake rate of the limiting nutrient by phytoplankton multiplied by the ratio of C:Limting Nutrient (e.g., N or P). Therefore, higher C:N and C:P ratios with higher temperature may indicate an increased potential for the export of organic carbon to the deep oceans for sequestration.

### 3.6 Comparison to other models and implication of the model results on the surface ocean

In this study, we have incorporated the effect of temperature on a macromolecular model of phytoplankton. We recognize there are other models that predict elemental stoichiometry, and some of these also include macromolecular allocation. These studies are reviewed in the publication of CFM-Phyto [19], and ones related to N_2_ fixing organisms are reviewed in [31]. Here, we also provide a review of these models in our supplementary material similar to [19]. We wish to point out the relatively small number of studies which consider macromolecular allocation, elemental stoichiometry, and the direct effect of temperature on these metrics. We selected these studies and made comparisons to our model (Table S4). These comparisons highlight the uniqueness of our model largely due to our use of laboratory results to constrain the model, resolving of more specific macromolecular pools for allocation, and prediction of resultant elemental stoichiometry of C:N:P (not only N:P or C:P).

To isolate the potential effect of temperature on the elemental stoichiometry, we have reflected our model results on the global ocean assuming temperature as the sole influence (Fig. S4). We recognize other factors influence the elemental stoichiometry, such as nutrient concentrations, and we may need further parameterization to represent diverse marine species. Input nutrient concentrations in culture experiments vary across studies [16], [38] and tend to be considerably higher than that of the marine environment, although the steady-state concentrations of growth-limiting nutrients in culture [60] may resemble those in marine [61]. Furthermore, more complete incorporation of the model into the ocean ecosystem may require coupling of nutrient uptake and macromolecular allocation. Despite that, our surface ocean simulation follows similar latitudinal patterns of previous works [17], [18], the largest values of N:P spanning from 20⁰ S to 20⁰N. Although our highest predicted N:P values (N:P = 45) are larger than the highest predicted values in other studies [17], [18], our N:P and C:P predictions are largely within the range of observation in marine phytoplankton and organic matter [10]. These similarities may suggest the strong influence of temperature on the marine elemental stoichiometry. Additionally, we provide predictions of a future ocean scenario in which the ocean surface warms by 4⁰C globally. We observe extension of high N:P values (N:P = 40–45) into higher latitudes. Also, there is a large decrease in N:C and P:C values, suggesting relative increase in C content exported to the deep ocean.

## 4 Conclusion

Our model resolves nutrient allocation in various macromolecular pools, including storage, for phytoplankton with increasing temperature, providing predictions and testable hypothesis for future experiments. Increasing temperature negatively affects nutrient dedication to biosynthesis, which, in turn, negatively affects the amount of molecules related to this process. The model result shows P-rich RNA and N-rich protein both decrease with temperature resulting in lower N:C and P:C at higher temperatures. However, RNA decreases more sharply than protein, resulting in a large decrease in P:C, and thus increased N:P values at increased temperatures. Additionally, our results show a higher allocation of carbon to storage with increasing temperature, which may contribute to increased export production. Our model results depict a similar pattern in the observed elemental stoichiometry across latitude when reflected on the surface ocean, suggesting macromolecular-mediated correlations between temperature and the elemental stoichiometry in the ocean.

## 5 Model availability

The model code for this study can be found in https://doi.org/10.5281/zenodo.5076472.

## Author contributions

**Gabrielle Armin:** Conceptualization, Methodology, Software, Formal analysis, Investigation, Writing-original draft, Writing- review & editing, Project administration. **Keisuke Inomura:** Conceptualization, Methodology, Resources, Writing- review & editing, Project administration, Funding acquisition.

## Declaration of Competing Interest

The authors declare that they have no known competing financial interests or personal relationships that could have appeared to influence the work reported in this paper.
