# Supplementary material for: Modeled temperature dependencies of macromolecular allocation and elemental stoichiometry in phytoplankton
Source: Comput Struct Biotechnol J. 2021 Sep 28;19:5421–7. doi: 10.1016/j.csbj.2021.09.028 (PMC8515405; doi:10.1016/j.csbj.2021.09.028)
Supplement: Supplementary data 1 [file mmc1.pdf]

A.1. Methods

We reduced the stoichiometric ratio of moles of nitrogen found in the chloroplasts to moles of chlorophyll carbon (mol-N/mol-C chl) from previous versions of the CFM-Phyto model to tailor the parameter towards *Chlamydomonas reinhardtii*. Using elemental ratios found in culture [1], we calculated this ratio through the following steps.

We normalized all molecular weights to carbon and calculated the weights (Table 1) based on supplementary material in a relevant laboratory study [1].

Table 1. Molecular weights of interest

	Molecular formula	g/C-mol
Biomass	$CH_{1.62}O_{0.41}N_{0.14}P_{0.01}S_{0.003}$	22.55
Protein	$CH_{1.6}O_{0.3}N_{0.27}S_{0.006}$	22.37
Chlorophyll	$CH_{1.3}O_{0.09}N_{0.07}$	15.72

We then determined the ratio of moles of protein per mole of biomass (mol p/mol b) using the values from Table 1 and the percent biomass composition found in culture.

$$\frac{40.23\ g\ CH_{1.6}O_{0.3}N_{0.27}S_{0.006}}{100\ g\ CH_{1.62}O_{0.41}N_{0.14}P_{0.01}S_{0.003}}\left(\frac{22.55\ g\ CH_{1.62}O_{0.41}N_{0.14}P_{0.01}S_{0.003}}{1\ mol\ CH_{1.62}O_{0.41}N_{0.14}P_{0.01}S_{0.003}}\right)\left(\frac{1\ mol\ CH_{1.6}O_{0.3}N_{0.27}S_{0.006}}{22.37\ g\ CH_{1.6}O_{0.3}N_{0.27}S_{0.006}}\right)$$

$$= 0.405\ \frac{mol\ CH_{1.6}O_{0.3}N_{0.27}S_{0.006}}{mol\ CH_{1.62}O_{0.41}N_{0.14}P_{0.01}S_{0.003}}$$

We assumed that half of the protein in the cell is comprised of the enzyme RuBisCO and halved this number. With that, 0.202 moles of protein would be considered to be found in the chloroplasts and, using the elemental ratio of N:C, 0.055 moles of nitrogen. In a similar manner, we calculated the moles of nitrogen in chlorophyll. We combined these values and divided by moles of carbon in chlorophyll. The resulting ratio was 0.81, which is lower than the model's

previous value. Therefore, we decided to lower this parameter to model the data more effectively.

Table 2. Compilation of elemental stoichiometry equations with temperature dependence

$$N{:}C = a_N\mu^2 + b_N\mu + c_N$$
where $$a_N = Y_{RNA}^{N:P}\left(\frac{A_{RNA}^{P}}{Arr}\right)\left(\left(\frac{A_{Bio}}{Arr}\right) + A_{Pho}A_{Chl}(I)\right)$$ $$b_N = \left(Y_{Chl}^{N:C}A_{Chl}(I) + Y_{Pro}^{N:C}\left(\left(\frac{A_{Bio}}{Arr}\right) + A_{Pho}A_{Chl}(I)\right) + Y_{RNA}^{N:P}\left(\frac{A_{RNA}^{P}}{Arr}\right)\left(A_{Pho}B_{Chl}(I) + Q_C^{Pro-Other}\right)\right)$$ $$c_N = Y_{Chl}^{N:C}B_{Chl}(I) + Y_{Pro}^{N:C}\left(A_{Pho}B_{Chl}(I) + Q_C^{Pro-Other}\right) + Y_{RNA}^{N:P}Q_{P,min}^{RNA} + Y_{DNA}^{N:C}Q_C^{DNA} + Q_N^{Sto}$$
$$P{:}C = a_P\mu^2 + b_P\mu + c_P$$
where $$a_P = \left(\frac{A_{RNA}^{P}}{Arr}\right)\left(\left(\frac{A_{Bio}}{Arr}\right) + A_{Pho}A_{Chl}(I)\right)$$ $$b_P = \left(\frac{A_{RNA}^{P}}{Arr}\right)\left(A_{Pho}B_{Chl}(I) + Q_C^{Pro-Other}\right) + A_{Pho}^{P:Chl}A_{Chl}(I)$$ $$c_P = Q_{P,min}^{RNA} + Y_{DNA}^{P:C}Q_C^{DNA} + A_{Pho}^{P:Chl}B_{Chl}(I) + Q_P^{Other0} + Q_P^{Sto}$$
$$N{:}P = \frac{N{:}C}{P{:}C}$$

See [2] detailed derivation.

Table 3. Description of equation parameters

Parameter	Definition	Units
A_{Bio}	Proportionality constant that relates growth rate to biosynthetic protein	mol C mol C^{-1} d
$A_{Chl}(I)$	The respiratory cost of synthesis divided by a given irradiance (I).	mol C mol C^{-1} d
A_{Pho}	Proportionality constant that relates cellular chlorophyll content to photosynthetic protein	mol C mol C^{-1}
$A_{Pho}^{P:Chl}$	Proportionality constant that relates cellular chlorophyll	mol P mol C^{-1}

	content to thylakoid phospholipids	
A_{RNA}^{P}	Proportionality constant that relates the total protein content and growth rate to the investment in RNA	mol P mol C^{-1} d
Arr	Reaction rate constant given by the Arrhenius formulation	dimensionless
$B_{Chl}(I)$	Ratio of the maintenance respiration rate to the per chlorophyll rate of photosynthesis at a given irradiance (I).	mol C mol C^{-1}
Q_{C}^{DNA}	Ratio of DNA in nucleic acids allocated to carbon pool	mol C mol C^{-1}
$Q_{C}^{Pro-Other}$	Ratio of proteins associated with essential metabolism allocated to carbon pool	mol C mol C^{-1}
Q_{N}^{Sto}	Nitrogen dedicated to storage	mol N mol C^{-1}
Q_{P}^{Other0}	Phosphorus distributed in a fixed pool consisting of non-photosynthetic phospholipids and associated with other molecules	mol P mol C^{-1}
$Q_{P,min}^{RNA}$	The minimum RNA that occurs at zero growth rate	mol P mol C^{-1}
Q_{P}^{Sto}	Phosphorus found in storage compounds including polyphosphate	mol P mol C^{-1}
μ	Growth rate	d^{-1}
$Y_{Chl}^{N:C}$	Constant elemental ratio for chlorophyll that relates nitrogen and carbon	mol N mol C^{-1}
$Y_{DNA}^{N:C}$	Constant elemental ratio for DNA that relates nitrogen and carbon	mol N mol C^{-1}
$Y_{DNA}^{P:C}$	Constant elemental ratio for DNA that relates phosphorus and carbon	mol P mol C^{-1}
$Y_{Pro}^{N:C}$	Constant elemental ratio for protein that relates nitrogen and carbon	mol N mol C^{-1}

$Y_{RNA}^{N:P}$	Constant elemental ratio for RNA that relates nitrogen and phosphorus	mol N mol P^{-1}

A.2. Results

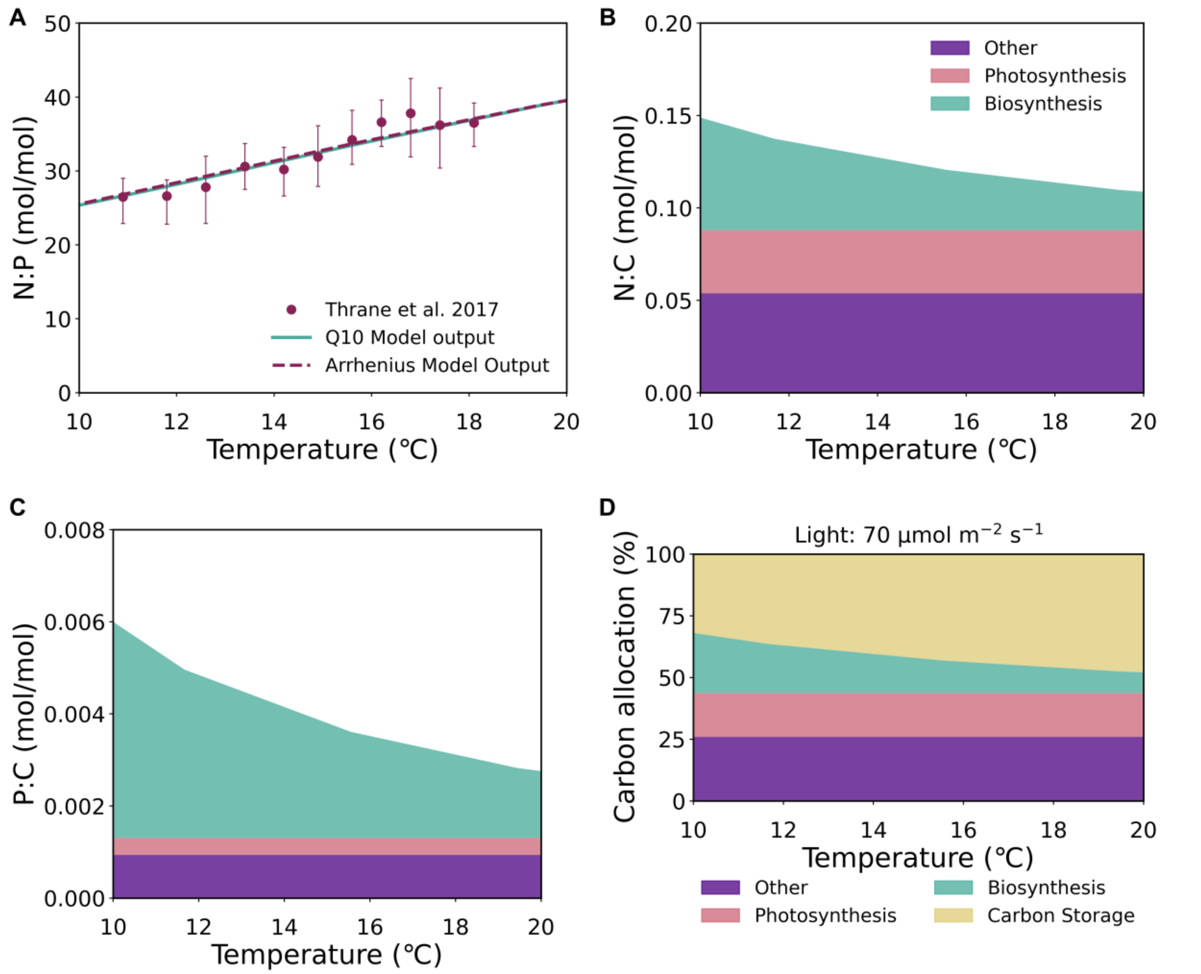

Figure S1. Modeled molar elemental ratios (N:P, N:C, P:C) under nutrient co-limitation over a range of temperatures in *Chlamydomonas reinhardtii* using the Q10 temperature formulation. Model produced N:P ratios (Panel A) for the Arrhenius and Q10 formulations were nearly identical to one another. Data points and error bars indicate average, upper, and lower values of N:P at each temperature [3]. N:C (Panel B) and P:C (Panel C) ratios also include respective N and P allocation to macromolecular pools of biosynthesis (teal area), photosynthesis (pink area), and other (purple area) over the temperature range. Carbon allocation percentage (Panel D) over a range of temperatures to four macromolecular pools: photosynthetic molecules, biosynthetic molecules, essential molecules ("Other"), and carbon storage.

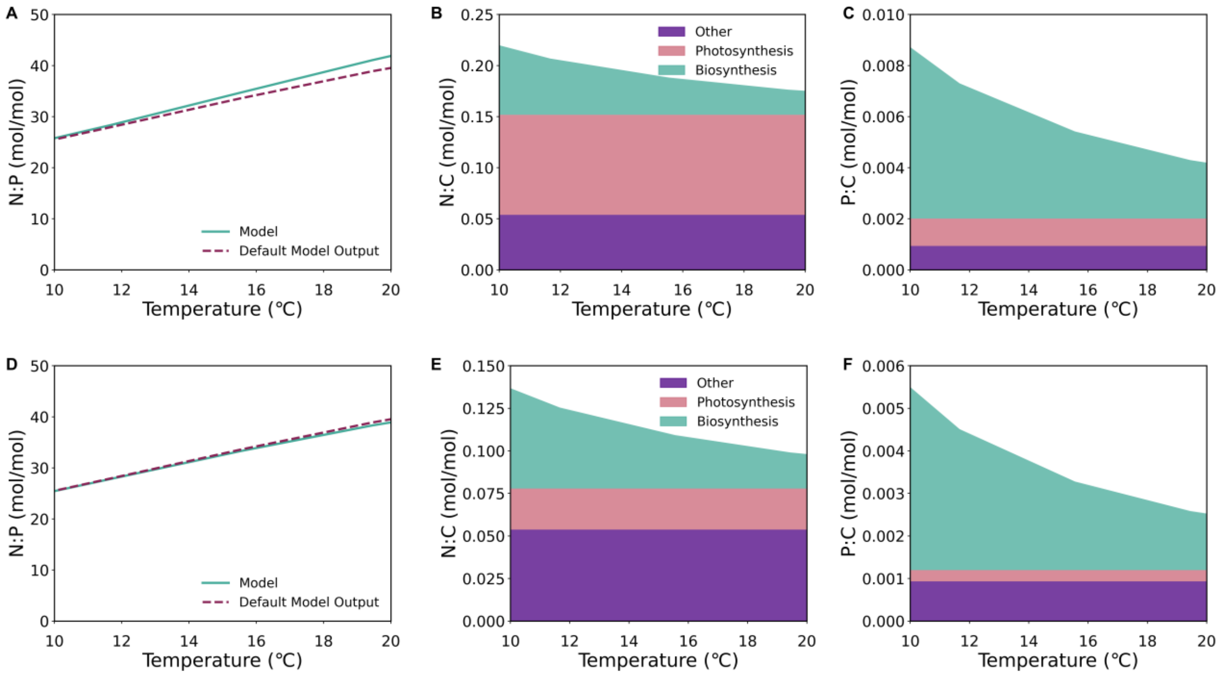

Figure S2. Modeled molar elemental ratios N:P (Panel A;D), N:C (Panel B;E), P:C (Panel C;F) under nutrient co-limitation over a range of temperatures for light intensities of 20 µmol photons $m^{-2}s^{-1}$ (Panels A-C) and 120 µmol photons $m^{-2}s^{-1}$ (Panels D-F). Default model output (dashed, maroon line) with dilution of 0.25 day^{-1} and 70 µmol photons $m^{-2}s^{-1}$ shown for comparison (Panel A;D). N:C (Panel B;E) and P:C (Panel C;F) ratios also include respective N and P allocation to macromolecular pools of biosynthesis (teal area), photosynthesis (pink area), and other (purple area) over the temperature range at these differing light intensities.

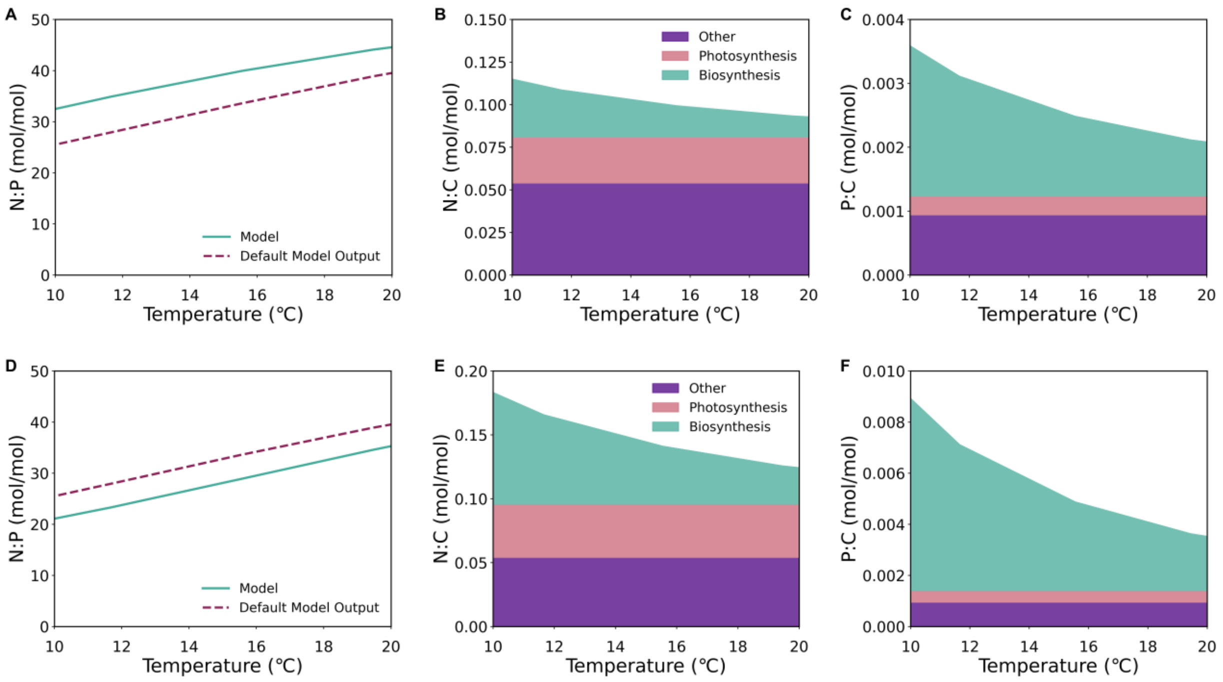

Figure S3. Modeled molar elemental ratios N:P (Panel A;D), N:C (Panel B;E), P:C (Panel C;F) under nutrient co-limitation over a range of temperatures for dilution rates of 0.15 day^{-1} (Panels A-C) and 0.35 day^{-1} (Panels D-F). Default model output (dashed, maroon line) with dilution of 0.25 day^{-1} shown for comparison (Panel A;D). N:C (Panel B;E) and P:C (Panel C;F) ratios also include respective N and P allocation to macromolecular pools of biosynthesis (teal area), photosynthesis (pink area), and other (purple area) over the temperature range at these differing growth rates.

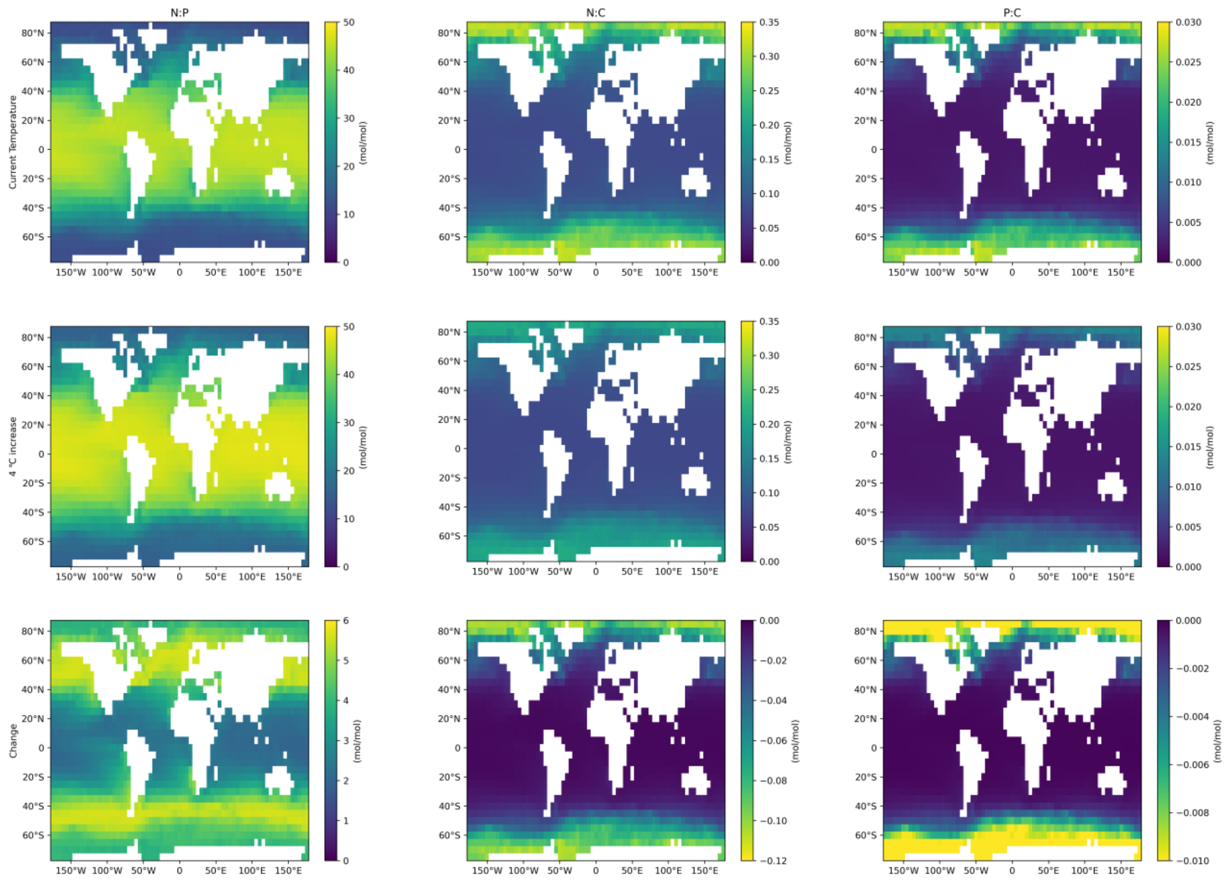

Figure S4. Modeled molar elemental ratios N:P (column 1), N:C (column 2), P:C (column 3) under nutrient co-limitation, saturating light (1000 µmol photons $m^{-2}s^{-1}$), and a growth rate of 0.25 day^{-1} for current ocean temperatures (row 1), future ocean temperatures (row 2), and the change between these two scenarios (row 3). "Business as usual" approach used for future ocean temperatures, which signifies a 4ºC increase globally. Darker colors signify lower values (rows 1-2) or less change (row 3). Temperature data from World Ocean Atlas [4].

A.3. Physiological Models of Phytoplankton

Here we review the physiological models of phytoplankton based the supplementary text of [2]. Physiological models of phytoplankton have evolved greatly over time, but the first mathematical representations of biological reactions still inform models today. These models began with focusing on a single limiting resource as the guiding factor in computing growth rates [5–7]. This was soon extended to multiple elemental pools [8,9], representations of

photoacclimation and temperature dependence [10], and to the “chain model” which quantified a relationship between intracellular fluxes of N and P [11].

Later models resolved allocation of carbon to specified macromolecular pools to maximize growth rate [12] which provided a link between elemental composition and cellular function [13]. This allowed following models to optimize resources and use provided constraints to determine the dependence of growth and stoichiometry on many environmental factors [14–24]. Further constraints on resource uptake versus allocation within the protein and resource pool [25,26] have been used in ocean models [27–30]. There are also models of nitrogen-fixing organisms that follow the concept of macromolecular allocation[31,32].

Simple models of heterotrophic bacteria calculate limits to growth under differing nutrient regimes using RNA/protein ratios and gene expression [33]. Another model of protein allocation in heterotrophic bacteria [34] evolved to include phototrophy [35] and saw comparable values of protein allocation to culture [36]. A coarse-grained allocation model for protein [37] was comparable to another laboratory study [38] and extended to simulate cellular growth and biomass production in a light limited chemostat [39].

Recently, genomics have proven to add complexity and detail to allocation models. This approach resolves thousands of cellular reactions [40] and can be constrained by allocation [41–43] or a desired outcome (e.g. growth).

Our model focuses on the temperature effect like some previously constructed models. Below, we outline the main differences between our model and other relevant models (Table S4).

Table S4. Comparison of our current work to recently published models of temperature dependence in phytoplankton physiology.

Author, Year	Manuscript Title	Key Points of Model	Armin and Inomura 2021
Geider, 1998 [10]	A dynamic regulatory model of phytoplanktonic acclimation to light, nutrients, and temperature	• Three indices of phytoplankton biomass: carbon, nitrogen, and chlorophyll a<br>• Predicts variability of N:C, Chl:N, and Chl:C in response to various nutrient and light regimes	• Elemental stoichiometry (N:P, P:C, N:C) and macromolecular allocation predictions<br>• Prediction includes phosphorus-related macromolecules and storage
Toseland, 2013 [15]<br><br>and<br><br>Daines, 2014 [16]	The impact of temperature on marine phytoplankton resource allocation and metabolism<br><br>Multiple environmental controls on phytoplankton growth strategies determine adaptive responses of the N:P ratio	• Resolves cell size and three sub-cellular allocation pools used: photosynthetic, biosynthetic, structure<br>• Q10 temperature formulation used<br>• Global predictions of average allocation to three compartments and N:P ratios in global ecosystem model	• Include storage of nutrients (C, N, P)<br>• Compare results of modeled N:P values to observed values in culture<br>• Arrhenius temperature formulation used<br>• Global predictions of N:P, P:C, and N:C<br>• Multiple nutrient limitation simulations provided
Garcia, 2020 [44]	Linking regional shifts in microbial genome adaptation with surface ocean biogeochemistry	• Predicts phytoplankton C:P ratios from temperature, irradiance, and metagenomic data<br>• Includes phosphorus luxury uptake pool	• Predict N:P, N:C, and P:C elemental ratios with temperature in three different nutrient regimes<br>• Include phosphorus, carbon, and nitrogen storage
Pahlow, 2020 [45]	Optimality-based non-Redfield plankton-ecosystem model (OPEM v1.1) in UVic-ESCM 2.9-Part 1: Implementation and model behaviour	• Global ecosystem model with variable C:N:P stoichiometry<br>• Allocation in phytoplankton comprised of light harvesting and nutrient acquisition<br>• Temperature dependencies follow Eppley curve	• Allocation to additional macromolecular pools<br>• Focus on phytoplankton alone, not observing community reactions. Want to understand the direct effect of temperature on physiology<br>• Arrhenius form used

A.4. References

1. Kliphuis AMJ, Klok AJ, Martens DE, Lamers PP, Janssen M, Wijffels RH (2012) Metabolic modeling of Chlamydomonas reinhardtii: Energy requirements for photoautotrophic growth and maintenance. J Appl Phycol. 24: 253–266.

2. Inomura K, Omta AW, Talmy D, Bragg J, Deutsch C, Follows MJ (2020) A Mechanistic Model of Macromolecular Allocation, Elemental Stoichiometry, and Growth Rate in Phytoplankton. Front Microbiol. 11: 1–22.

3. Thrane JE, Hessen DO, Andersen T (2017) Plasticity in algal stoichiometry: Experimental evidence of a temperature-induced shift in optimal supply N:P ratio. Limnol Oceanogr. 62: 1346–1354.

4. NOAA (2018) WOA 2018-Data Access: Statistical mean of temperature on 5° grid for all decades. In: World Ocean Atlas.

5. Monod J (1949) The Growth of Bacterial Cultures. Annu Rev Microbiol. 3: 371–394.

6. Caperon J (1968) Population Growth Response of Isochrysis Galbana to Nitrate Variation at Limiting Concentrations. Ecology. 49: 866–872.

7. Droop MR (1968) Vitamin B12 and marine ecology. IV. The kinetics of uptake, growth, and inhibition. Monochrysis Lutheri J Mar Biol Assoc United Kingdom. 48: 689–733.

8. Flynn KJ (2008) The importance of the form of the quota curve and control of non-limiting nutrient transport in phytoplankton models. J Plankton Res. 30: 423–438.

9. Ågren GI (2004) The C:N:P stoichiometry of autotrophs - Theory and observations. Ecol Lett. 7: 185–191.

10. Geider RJ, MacIntyre HL, Kana TM (1998) A dynamic regulatory model of phytoplanktonic acclimation to light, nutrients, and temperature. Limnol Oceanogr. 43: 679–694.

11. Pahlow M, Oschlies A (2009) Chain model of phytoplankton P, N and light colimitation. Mar Ecol Prog Ser. 376: 69–83.

12. Shuter B (1979) A model of physiological adaptation in unicellular algae. J Theor Biol. 78: 519–552.

13. Sterner RW, Elser JJ (2002.) Ecological Stoichiometry: the Biology of Elements from Molecules to the Biosphere.

14. Klausmeier CA, Litchman E, Daufreshna T, Levin SA (2004) Optimal nitrogen-to-phosphorus stoichiometry of phytoplankton. Nature. 429: 171–174.

15. Toseland A, Daines SJ, Clark JR, Kirkham A, Strauss J, Uhlig C, et al. (2013) The impact of temperature on marine phytoplankton resource allocation and metabolism. Nat Clim Chang. 3: 979–984.

16. Daines SJ, Clark JR, Lenton TM (2014) Multiple environmental controls on phytoplankton growth strategies determine adaptive responses of the N:P ratio. Ecol Lett. 17: 414–425.

17. Talmy D, Blackford J, Hardman-Mountford NJ, Dumbrell AJ, Geider RJ (2013) An optimality model of photoadaptation in contrasting aquatic light regimes. Limnol Oceanogr. 58: 1802–1818.

18. Flynn KJ (2001) Temperature Interactions in Phytoplankton. J Plankton Res. 23: 977–997.

19. Flynn KJ (2005) Modelling marine phytoplankton growth under eutrophic conditions. J Sea Res. 54: 92–103.

20. Ghyoot C, Flynn KJ, Mitra A, Lancelot C, Gypens N (2017) Modeling plankton mixotrophy: A mechanistic model consistent with the shuter-type biochemical approach. Front Ecol Evol. 5: 1–16.

21. Moreno AR, Hagstrom GI, Primeau FW, Levin SA, Martiny AC (2018) Marine phytoplankton stoichiometry mediates nonlinear interactions between nutrient supply, temperature, and atmospheric CO2. Biogeosciences. 15: 2761–2779.

22. Nicholson DP, Stanley RHR, Doney SC (2018) A Phytoplankton Model for the Allocation of Gross Photosynthetic Energy Including the Trade-Offs of Diazotrophy. J Geophys Res Biogeosciences. 123: 1796–1816.

23. Talmy D, Blackford J, Hardman-Mountford NJ, Polimene L, Follows MJ, Geider RJ (2014) Flexible C : N ratio enhances metabolism of large phytoplankton when resource supply is intermittent. Biogeosciences. 11: 4881–4895.

24. Clark JR, Lenton TM, Williams HTP, Daines SJ (2013) Environmental selection and resource allocation determine spatial patterns in picophytoplankton cell size. Limnol Oceanogr. 58: 1008–1022.

25. Bonachela JA, Allison SD, Martiny AC, Levin SA (2013) A model for variable phytoplankton stoichiometry based on cell protein regulation. Biogeosciences. 10: 4341–4356.

26. Smith SL, Yamanaka Y, Pahlow M, Oschlies A (2009) Optimal uptake kinetics:

Physiological acclimation explains the pattern of nitrate uptake by phytoplankton in the ocean. Mar Ecol Prog Ser. 384: 1–12.

27. Smith SL, Pahlow M, Merico A, Acevedo-Trejos E, Sasai Y, Yoshikawa C (2016) Flexible phytoplankton functional type (FlexPFT) model: Size-scaling of traits and optimal growth. j Plankt Res. 38: 977–992.

28. Chen B, Lan Smith S (2018) CITRATE 1.0: Phytoplankton continuous trait-distribution model with one-dimensional physical transport applied to the North Pacific. Geosci Model Dev. 11: 467–495.

29. Chen B, Smith SL (2018) Optimality-based approach for computationally efficient modeling of phytoplankton growth, chlorophyll-to-carbon, and nitrogen-to-carbon ratios. Ecol Modell. 385: 197–212.

30. Chen B, Smith SL, Wirtz KW (2019) Effect of phytoplankton size diversity on primary productivity in the North Pacific: trait distributions under environmental variability. Ecol Lett. 22: 56–66.

31. Inomura K, Masuda T, Gauglitz JM (2019) Active nitrogen fixation by Crocosphaera expands their niche despite the presence of ammonium – A case study. Nat Sci Reports. 9: 1–11.

32. Luo YW, Shi D, Kranz SA, Hopkinson BM, Hong H, Shen R, et al. (2019) Reduced nitrogenase efficiency dominates response of the globally important nitrogen fixer Trichodesmium to ocean acidification. Nat Commun. 10: 1–12.

33. Scott M, Gunderson CW, Mateescu EM, Zhang Z, Hwa T (2010) Interdependence of Cell

Growth and Gene Expression: Origins and Consequences. Science. 330: 1099–1102.

34. Molenaar D, Van Berlo R, De Ridder D, Teusink B (2009) Shifts in growth strategies reflect tradeoffs in cellular economics. Mol Syst Biol. 5: 1–10.

35. Burnap RL (2015) Systems and photosystems: Cellular limits of autotrophic productivity in cyanobacteria. Front Bioeng Biotechnol. 3: 1–13.

36. Jahn M, Vialas V, Karlsen J, Maddalo G, Edfors F, Forsström B, et al. (2018) Growth of Cyanobacteria Is Constrained by the Abundance of Light and Carbon Assimilation Proteins. Cell Rep. 25: 478-486.e8.

37. Faizi M, Zavřel T, Loureiro C, Červený J, Steuer R (2018) A model of optimal protein allocation during phototrophic growth. BioSystems. 166: 26–36.

38. Zavřel T, Faizi M, Loureiro C, Poschmann G, Stühler K, Sinetova M, et al. (2019) Quantitative insights into the cyanobacterial cell economy. Elife. 8: 1–29.

39. Faizi M, Steuer R (2019) Optimal proteome allocation strategies for phototrophic growth in a light-limited chemostat. Microb Cell Fact. 18: 1–18.

40. Orth JD, Thiele I, Palsson BO (2010) What is flux balance analysis? Nat Biotechnol. 28: 245–248.

41. Müller S, Regensburger G, Steuer R (2015) Resource allocation in metabolic networks: Kinetic optimization and approximations by FBA. Biochem Soc Trans. 43: 1195–1200.

42. Rugen M, Bockmayr A, Steuer R (2015) Elucidating temporal resource allocation and diurnal dynamics in phototrophic metabolism using conditional FBA. Sci Rep. 5: 1–16.

43. Reimers AM, Knoop H, Bockmayr A, Steuer R (2017) Cellular trade-offs and optimal

resource allocation during cyanobacterial diurnal growth. Proc Natl Acad Sci U S A. 114: E6457–E6465.

44. Garcia CA, Hagstrom GI, Larkin AA, Ustick LJ, Levin SA, Lomas MW, et al. (2020) Linking regional shifts in microbial genome adaptation with surface ocean biogeochemistry. Philos Trans R Soc B Biol Sci. 375.

45. Pahlow M, Chien C Te, Arteaga LA, Oschlies A (2020) Optimality-based non-Redfield plankton-ecosystem model (OPEM v1.1) in UVic-ESCM 2.9 - Part 1: Implementation and model behaviour. Geosci Model Dev. 13: 4663–4690.
